# Use of over-the-counter analgesics in Norwegian children – a national cross-sectional study

**DOI:** 10.1177/14034948251328492

**Published:** 2025-03-25

**Authors:** Frøydis Enstad, Sølvi Helseth, Borghild Løyland, Kristin Haraldstad, Siv Olga Skarstein

**Affiliations:** 1 Norwegian Social Research (NOVA), Oslo Metropolitan University, Oslo, Norway; 2 Department of Nursing and Health Promotion, Oslo Metropolitan University, Oslo, Norway; 3 Department of Health and Nursing Science, University of Agder, Kristiansand, Norway

**Keywords:** Over-the-counter analgesics, children, recurrent pain, depressive symptoms, bullying, population-based sample

## Abstract

**Aims:**

We aimed to examine the proportion of recent use of over-the-counter (OTC) analgesics among Norwegian children aged 10 to 12, and explore the relationship between a wide array of factors, both within and outside the indications for use of the medication and recent use of OTC analgesics in this age group.

**Methods:**

Data were drawn from the nationwide, population-based Ungdata Junior study in Norway, encompassing children aged 10 to 12 (*N* = 102,919). We examined factors both within (recurrent pain in different parts of the body) and beyond (repeated bullying, depressive symptoms, screen time and organised activity frequency) medication indications by means of logistic regression. Covariates and background variables included sleep duration, frequency of sports activities, gender, age and socioeconomic status.

**Results:**

The results revealed a high prevalence of recent OTC analgesic use (23.7%). Associations were noted with factors both within and beyond the medication’s indications for use. Specifically, adjusted for all variables, recurrent headache (OR = 2.93, 95% CI = 2.81–3.05) and pain (OR = 1.16, 95% CI = 1.11–1.21), depressive symptoms (OR = 1.19, 95% CI = 1.15–1.22) and repeated bullying (OR = 1.07, 95% CI = 1.02–1.14) were significantly linked with an increased risk of recent OTC analgesic use.

**Conclusions:**

**Our findings suggest a potential misuse of OTC analgesics early in life and raise concerns about potential overuse and unhealthy coping strategies. Enhancing children’s and parents’ understanding of pain and stress management may improve health behaviours and mitigate potential adverse effects from OTC analgesic use.**

## Background

The use of over-the-counter (OTC) analgesics, also named non-prescription analgesics, is increasing in the general population [1]. Recent Norwegian prevalence studies furthermore indicate that the use of non-prescription painkillers in the child and youth population is high and increasing [2-5]. In Norway, as in the rest of Europe, the most used OTC analgesics are paracetamol (acetaminophen), under brands such as Panodil, Pinex and Paracet, followed by the non-steroidal anti-inflammatory drug (NSAID) group contained in brands such as Ibux, Brufen and Ibumetin [6]. In most Nordic countries, OTC analgesics are legally available without a prescription or advice from health professionals and can be purchased in pharmacies, supermarkets, and petrol stations [6]. It is worth noting that sales of non-prescription paracetamol in Norway have increased by 15.44% over the past decade (from a 14.9 defined daily dose/1000 inhabitants/day in 2014 to 17.2 in 2023) [6, 7].

Paracetamol is recommended by Norwegian health authorities as the first choice in treating mild to moderate pain (e.g. headaches, menstrual pain, pain in muscles and joints, and fever [6]). Recommended use of OTC analgesics is considered relatively harmless [8]. Nevertheless, inappropriate, excessive or regular use of OTC analgesics has been associated with medication-overuse headaches and may cause serious and irreversible damage to the kidneys and liver [9]. NSAIDs can furthermore cause severe gastrointestinal ulceration, bleeding and renal and cardiac complications, even when taken as recommended [10]. Also of concern is the reported increase in paracetamol-linked deaths, suicide attempts and intended overdose among young people [11]. Moreover, the majority of medication poisonings in children and adolescents, which have been on the rise in recent decades, can be attributed to a high intake of easily available OTC analgesics [12]. To reduce the risk of misuse and overdose, pharmacies and grocery stores in Norway can only sell one packet of OTC analgesics at a time. Nonetheless, the Norwegian authorities are concerned about young people’s use of painkillers and have recently launched an information campaign on the government’s official information platform for young people, which encourages both less and the correct use of paracetamol [13].

Most children and adolecents have access to OTC analgesics at home, and self-administration of these drugs often begins in early adolescence [14]. Studies focusing on adolescents have found that while most exhibit a responsible attitude toward the use of OTC analgesics, a subset demonstrates a more careless pattern of use [15]. A systematic review of self-medication practices among adolescents aged 13 to 18 years uncovered a concerning pattern of misuse, including the frequent use of OTC analgesics beyond the recommended dosage, alongside a general lack of knowledge about the analgesics [16]. In Norway, the prevalence of past-week analgesic use ranges from 24% to 26% [4, 5]. Given the high prevalence of self-reported use of painkillers, even in the childhood years, there is growing concern over the potentially inappropriate use of OTC analgesics during the childhood and adolescent life stages, with the inherent risk of overuse. Consequently, it appears vital to examine the factors associated with the use of OTCs, both within and beyond the intended range of medication use, especially among children, where knowledge is still limited.

Studies of factors within the indications for use of OTC analgesics generally show that pain from several parts of the body is found to be a strong predictor of use among adolescents, however headace is the most common reason for the intake of such medicines [5, 16]. In general, more girls than boys have persistent pain and use such medicines more frequently [5, 17]. Girls might furthermore have primary dysmenorrhoea, and in such cases, OTC analgesics are the recommended medical treatment [18]. Not surprisingly, therefore, frequent use of OTC analgesics is more common among girls [5, 16, 19]. However, a recent study has found that a considerable part of the observed gender difference in OTC analgesic usage can be traced back to differences in the frequency and severity of physical and mental health problems [19].

Although pain is the factor most consistently associated with the use of OTC analgesics, several studies have found that children’s and adolescents’ use of OTC analgesics is associated with factors well outside of the range of the medication. A Norwegian cross-sectional study among adolescents (15–16 years) found that those who frequently used OTC analgesics not only experienced more pain, but also slept less, had lower self-esteem, lower school attendance, less ambitious educational plans, and engaged more often in binge drinking compared to those who seldom or never used such medications [20]. Moreover, a Danish study of adolescents with mean ages 11.6 to 15.6 years found that victimisation from bullying was associated with use of OTC analgesics, even when controlling for the higher prevalence of symptoms among bullied victims [21]. Along the same lines, several studies have found that trauma-exposed children and adolescents in Norway use OTC analgesics for headaches and musculoskeletal pain more frequently than those not exposed to trauma [5, 22]. Other studies have also demonstrated associations between mental health problems and psychological distress, such as anxiety and depression, and the use of OTC analgesics [5, 19, 23]. In sum, while pain is undoubtedly a key driver of OTC analgesic use in adolescence, a variety of factors well beyond the realm of physical discomfort play a role. The findings point to a complex interplay between physical pain, negative life events and psychological distress or mental health problems.

There is furthermore a prevailing concern that today’s childhood is characterised by increasing screen time, poor sleep hygiene and mounting pressure to perform in various areas, such as school, sports and other leisure activities. It is therefore important to examine more systematically whether such life strains drive the use of OTC analgesics in this age group, as has been suggested elsewhere [24]. It has for example been suggested that pain induced by physical training load is a factor associated with the use of OTC among adolescents [25]. Whether this is also the case among children remains unknown.

As the above-mentioned studies are mainly limited to adolescents from the age of 13 years, little is known about the use of OTC analgesics in the childhood life phase. This study aims to address this knowledge gap by examining the degree to which factors within and outside of the indications for use of OTC analgesics is associated with recent use of OTC analgesics among children. In the present study, we used data from a nationally representative population-based sample of Norwegian children (aged 10 to 12) to examine: i) the proportion of recent use of OTC analgesics, ii) which factors within and outside the indications for use of OTC analgesics are associated with recent use of OTC analgesics, and iii) examine whether these relationships remain upon adjustment for each other, as well as with covariates and background variables, such as hours of sleep, sports activity frequency, gender, age, socioeconomic status and year of data collection.

## Methods

### Study design and setting

The sample was drawn from the Norwegian nationwide Ungdata Junior surveys available for grades 5 to 7 in elementary school (ages 10 to 12) [26]. Ungdata Junior is a school-based electronic survey, covering a wide range of topics, such as school, physical and mental health, family and friends, leisure activities and well-being. The survey is offered to all municipalities in Norway and Svalbard (Spitsbergen) free of charge. Each municipality is recommended to carry out a survey every 3 years. Municipalities in different regions around Norway carry out the survey in different years. Over a 3-year period, a nationally representative sample is obtained.

We used data from children in grades 5 to 7 participating in the years 2020, 2021 and 2022, covering birth cohorts born from 2007 to 2011. In this age group, 99% of the population attend elementary school. Private and public schools from a total of 199 municipalities spread across Norway and Svalbard participated in the study. The children completed the electronic questionnaire during one school lesson, supervised by classroom teachers. The overall response rate was 77% (*N* = 105,560). The response rate per year was 52% (*N* =10,997) in 2020, 82% in 2021 (*N* = 58,450) and 84% (*N* = 41,356) in 2022. In 2020, many of the participating schools were forced to stop the implementation of the study due to the COVID pandemic. Some of the municipalities who were meant to carry out the study in 2020, therefore, chose to join again the following year. The schools that participated again in 2021 were removed from the 2020 sample so that no child was represented twice in the drawn sample (5243 respondents were removed from the 2020 sample). Participants in the different grades were equally distributed. All participants and their parents or legal guardians were informed that participation in the Ungdata Junior survey was voluntary. Parents and legal guardians could withdraw their children from participation. The study design has been described in detail elsewhere [26].

The analyses in the present study were based on a sample of children who answered the question about use of OTC analgesics (*N* = 102,919). Of all respondents, 2641 (2.5%) did not answer this question.

### Measures

*Recent use of OTC analgesics* was measured by asking ‘Have you used tablets for headaches or other pain in the past week?’, with response options ‘No’ (coded 0) and ‘Yes’ (coded 1).

#### Variables within the indications for use of OTC analgesics

*Information about headache and pain* was obtained by asking ‘Have you had any of these ailments in the past month?’ and respondents could indicate the degree of ailment for headache, pain in the neck or shoulders and pain in the stomach with response options ‘Never’, ‘Sometimes’, ‘Several times’, and ‘Daily’. In alignment with research in the field of physical health complaints in children and adolescents, these indicators were dichotomised into recurrent ailments (‘Several times’ and ‘Daily’) and non-recurrent ailments (‘Never’ and ‘Sometimes’) [27].

#### Variables outside of the indications for use of OTC analgesics

*Repeated bullying* was measured by asking ‘Think about the past few months. Have you been excluded, bullied, or threatened by other children at school or in your leisure time?’, with six response options ranging from ‘Never’ to ‘Yes, several times a week’. We chose to focus on bullying with a certain regularity, as this provides a better indication of the degree of severity and eases interpretation of the results. As such, the variable was recoded into a dummy variable where those experiencing bullying never, almost never or about once a month (value 0) were contrasted with those experiencing bullying once a fortnight or more often (value 1). *Depressive symptoms* were assessed using six items derived from the Low Mood (major depressive disorder) subscale, which is a component of the Revised Child Anxiety and Depression Scale from the American Psychiatric Association [28]. The items evaluate feelings of joylessness, sleep disturbances, low energy levels, sensations of sadness or emptiness, difficulties with clear thinking and feelings of low self-worth. Items were rated on a 4-point Likert scale from 1 (Never) to 4 (Very often) and mean scores were computed, where only respondents answering at least three items were included in the score. Cronbach’s alpha was .84, indicating good internal consistency. The children provided information about *screen time* outside of school hours (response options ranging from no time to more than 6 h) and recoded into a dummy variable (≤3 h per day coded 0, and ≥3 h per day coded 1). Previous studies of Norwegian adolescents have used this cut-off value for screen time [29]. Lastly, the children were asked to specify the number of weeknights (from Monday and Friday) they participate in organised leisure activities. The response options ranged from 0, indicating ‘No nights’ to 5, representing ‘Five nights a week’.

### Covariates and background variables

*Hours of sleep* was measured by asking the respondents to indicate how many hours of sleep they had in the previous night. Response options ranged from 1 to 7, with 1 indicating the lowest level of sleep (6 h or less) and 7 indicating the highest amount of sleep (12 h or more). Lastly, the respondents were asked to indicate how many nights a week they take part in sports activities outside of school. The answers were given on a frequency scale ranging from 0 (‘Never’) to 5 (‘Every day’). *Gende*r (‘boy’ coded 0, ‘girl’ coded 1) was recorded. Grades were recorded as a proxy for *age*. Average scores on the four-item Family Affluence Scale II [30] were used to measure *socioeconomic status*. The scale includes items assessing the number of computers, tablets, and cars in the family, how many times the family had gone on holiday within the past year, and whether the respondents had their own room at home. Finally, the *year of data collection* was recorded.

### Statistical analysis

The prevalence of recent (use in the past week) and non-recent use (not used in the past week) of OTC analgesics are described. The relationship between the potential explanatory variables and recent use of OTC analgesics at ages 10 to 12 was examined by means of logistic regression analyses. The variables in the models were categorised under two domains: within the indications for use of OTC analgesics (headache, pain in the neck and shoulders and stomach ache) and factors outside the indications for use (experienced bullying, depressive symptoms, screen time and frequency of organised activity participation). Covariates and background variables included hours of sleep, frequency of sports activities, gender, age, socioeconomic status and year of data collection. Firstly, all variables within and outside the indications for use were evaluated one by one in a series of bivariate logistic regression analyses. Non-recent use of OTC analgesics was chosen as the comparison group. The analysis produced the odds of recent use of OTC analgesics compared to non-recent use. Secondly, we entered all variables within and outside the indications for use simultaneously into a multivariable regression analysis. Thirdly, we adjusted all variables for the covariates and background variables. Across the 13 variables that were used in the analyses, the proportion of missing data ranged from 0.6% for gender to 4.3% for grade level. Listwise deletion was used to handle missing data and is considered appropriate under conditions where the lack of data shows no particular pattern, and when the sample size is of a considerable size such that sufficient power is likely to remain for detecting meaningful effects [31]. To verify the robustness of our results, all analyses were additionally re-run using pairwise deletion to handle missing data. The results were largely the same across approaches. To reduce risks related to multiple testing and risk of Type I errors, we set the level of significance at *p* < 0.01. Data were analysed using IBM SPSS v.27.

## Results

Table I provides the descriptive statistics for the study variables. As shown, 23.7% of the children reported recent use of OTC analgesics. 17.8% reported recurrent headaches, 23.0% reported recurrent pain in the neck or shoulders and 19.8% reported recurrent stomach ache. Some 10.7% of the children reported experiencing repeated bullying. On average, the respondents reported a score of 1.8 (SD = 0.6, range 1–4) for depressive symptoms, indicating that most of the children were on the lower end of the scale. Boys and girls, and the different age groups, were equally distributed. The results furthermore showed a mean score of 2.5 (SD = 0.5, range 0–3) on the socioeconomic status variable, indicating that most of the study participants were in the higher range.

**Table I.** Descriptive statistics of all variables under study.

	*M*	SD	Range
Recent use of OTC analgesics^a^	23.7%		
Within indications for use of OTC			
Recurrent headache	17.8%		
Recurrent pain in the neck or shoulders	23.0%		
Recurrent stomach ache	19.8%		
Outside indications for use of OTC			
Repeated bullying	10.7%		
Depressive symptoms	1.8	0.6	1–4
Screen time (≥ 3 h per day)	49.4%		
Organised activity frequency	2.0	1.6	0–5
Covariates and background variables			
Hours of sleep	3.7	1.4	1–7
Sports activity frequency	2.3	1.4	0–5
Female	49.3%		
5^th^ grade (10 years)	31.0%		
6^th^ grade (11 years)	32.0%		
7^th^ grade (12 years)	32.8%		
Socioeconomic status	2.5	0.5	0–3
Participants in year 2020	5.5%		
Participants in year 2021	55.4%		
Participants in year 2022	39.1%		

*Note. M* = mean; SD = standard deviation.

a Recent use of OTC analgesics was measured by asking about the past week. Other variables, both within and outside the indications, were measured using different time frames. Please refer to the methods section for further details.

Table II presents the logistic regression models predicting recent use of OTC analgesics, with non-recent use serving as the reference category. In Model 1 (bivariate analyses), variables both within and outside the indications for use were associated with recent use of OTCs. For example, recurrent headache (within the indications for use) was significantly associated with OTC use (OR = 3.95, 95% CI = 3.80–4.07, *p* < 0.001) indicating that individuals who experienced headaches often or very often were approximately four times more likely to have used OTCs recently compared to those who experienced headache more seldom. Other significant predictors within the indications for use included recurrent pain in the neck or shoulders, stomach ache, and as factors outside of the indications for use repeated bullying, depressive symptoms and screen time. In Model 2 (multivariable analysis), all these variables were included simultaneously. Most associations remained significant but were generally attenuated. For example, the OR for recurrent headache decreased to 2.98 (95% CI = 2.86–3.10, *p* < 0.001). In Model 3 (adjusted model), all variables were adjusted for covariates, background variables and year of data collection. The associations within the indications for use generally remained similar to Model 2, indicating that these factors were significant predictors of OTC use even after adjustment. For instance, recurrent headache remained a significant predictor (OR = 2.93, 95% CI = 2.81–3.05, *p* < 0.001). Furthermore, outside the indications for use, depressive symptoms (OR =1.19, CI = 1.15–1.22, *p* < 0.001) and repeated bullying (OR = 1.07, CI = 1.02–1.14, *p* < 0.01) significantly increased the risk of recent use. Moreover, being female significantly increased the odds of recent OTC use by 1.09 (95% CI = 1.06–1.13, *p* < 0.001). Also, notably, OR for year of data collection was 1.19 (95% CI = 1.15–1.22, *p* < 0.001), which could suggest a possible increase in OTC use over the years. However, given the uneven distribution of respondents across the 3 years, this result needs to be interpreted with caution. Overall, the models showed that both variables within the indications for use (like recurrent headache and pains) and outside the indications for use (like repeated bullying and depressive symptoms) significantly increased the odds of recent OTC use. Also of note, a higher frequency of participation in sports significantly increased the odds of recent use of OTC analgesics (OR = 1.07, CI = 1.06–1.09, *p* < 0.001).

**Table II.** Logistic regression models with recent use of OTC analgesics as the dependent variable, and no recent use of OTC analgesics as the reference category.

	Recent use of OTC analgesics		
	Model 1	Model 2	Model 3
	OR (95% CI)	OR (95% CI)	OR (95% CI)
Within indications for use of OTC			
Recurrent headache	3.95 (3.80–4.07)**	2.98 (2.86–3.10)**	2.93 (2.81–3.05)**
Recurrent pain in the neck or shoulders	1.92 (1.86–2.00)**	1.16 (1.12–1.21)**	1.16 (1.11–1.21)**
Recurrent stomach ache	2.43 (2.36–2.52)**	1.44 (1.38–1.50)**	1.43 (1.37–1.49)**
Outside indications for use of OTC			
Repeated bullying	1.64 (1.56–1.71)**	1.06 (1.01–1.12)	1.07 (1.02–1.14)*
Depressive symptoms	1.80 (1.76–1.84)**	1.19 (1.16–1.23)**	1.19 (1.15–1.22)**
Screen time ≥ 3 hours per day	1.23 (1.20–1.27)**	1.02 (0.99–1.05)	1.03 (1.00–1.07)
Organised activity frequency	0.95 (0.94–0.96)**	0.98 (0.97–0.99)**	0.95 (0.94–0.96)
Covariates and background variables			
Hours of sleep			0.99 (0.98–1.01)
Sports activity frequency			1.07 (1.06–1.09)**
Gender (girl = 1)			1.09 (1.06–1.13)**
Age			1.02 (1.00–1.04)
Socioeconomic status			1.00 (1.00–1.01)
Year of data collection			1.19 (1.15–1.22)**

*Note*. OR = odds ratio; 95% CI = 95% confidence interval of OR.

Model 1: bivariate analyses were conducted for each variable within and outside the indications for use of OTC; Model 2: all variables within and outside the indications for use of OTC were included simultaneously; and Model 3: all variables adjusted for covariates and background variables.

* *p* < 0.01; **p < 0.001.

## Discussion

In this study, recent use of OTC analgesics was identified in 23.7% of children aged 10 to 12 years. We identified factors that were significantly associated with recent use of OTCs both within and outside the range of indications for use of such medications. Not surprisingly, the strongest and most consistent effect was found for recurrent headache, but also recurrent pain in different parts of the body was consistently and significantly related to increased risk of recent use. Moreover, consistent results were found for different life strains, where depressive symptoms and having experienced repeated bullying were significantly related to increased risk of recent use of OTC analgesics. The results also showed that girls had a slightly higher risk of recent use of OTC analgesics compared to boys.

Overall, our findings are well in line with previous studies in that the strongest explanatory factor for use of OTC analgesics was headache, and that recent use of OTC analgesics were also related to factors other than pain [5, 16, 32]. As most of the previous studies are on adolescents, this study adds to the knowledge by demonstrating that among children, a complex set of factors are associated with recent use of OTC analgesics. This poses a potential risk of inappropriate use of medications and may initiate unhealthy coping strategies and risky health behaviour during adolescence and into adulthood.

The prevalence of headaches in children and adolescents has been increasing in recent decades [33]. The prevalence of pain varies across studies, but a systematic review revealed a high and increasing prevalence of self-reported pain among adolescents across 42 countries [34]. The high prevalence of pain among children and adolescents may suggest a high potential use of OTC analgesics. Nonetheless, the high prevalence of recent use of OTC analgesics among 10 to 12 years olds is surprising, and higher than reports in adolescents [2]. Although there are no age restrictions for purchasing OTC analgesics at the pharmacy in Norway (outside the pharmacy the minimum age is 18), there is reason to presume that most children access OTC analgesic in their home environment [16]. Studies have shown that parents are the most important source of information regarding the use of OTC analgesics and that parents are their main suppliers [16]. In Norway, the general sales of both OTC analgesics and prescription pain medicine have increased [6, 7]. This general increase observed in the use of pain medicines could mean that more adults in a parental role use such medicines and that this behaviour is transferred to their children directly (by parents supply their children with medicines) and/or indirectly (by children copying parental behaviour). However, further research is needed to establish whether there has been a general change in behaviours and attitudes towards the use of OTC analgesics that could explain the high prevalence of use in childhood.

Interestingly, our study revealed that children who experience repeated bullying or report higher levels of depressive symptoms are at an increased risk of recent OTC analgesic use. It is well-documented that stressors like bullying and psychological difficulties correlate with physical pain [21]. However, our results suggest that bullying and psychological distress may act as unique risk factors for OTC analgesic use, beyond the mere experience of pain. This finding aligns with previous studies that have identified a strong link between bullying and violence and increased OTC analgesic use [5, 21]. Similarly, it has been found that experiencing interpersonal violence in childhood corresponds with increased use of OTC analgesics to address musculoskeletal pain and headaches in young adulthood [22].

Assuming parents are the primary suppliers of OTC analgesics, our findings underscore the need for parental guidance on identifying causes of childhood distress and instilling appropriate coping strategies. Late and middle childhood, along with early adolescence, are periods characterised by transitions and events that can induce psychological distress. Additionally, children may manifest psychological distress through somatic complaints [8]. If OTC analgesics are used to alleviate symptoms of psychosocial difficulties or psychological distress, this not only increases the risk of overuse but may also mask serious psychosocial challenges that children and parents struggle to articulate [8]. Furthermore, research suggests that family methods of coping with pain are evident in adolescents’ use of OTC analgesics, and adolescents learn pain management and coping strategies from their parents [10]. Moreover, mothers with chronic pain are five times more likely to report frequent pain in their children compared to mothers without pain [35]. This suggests that maternal health factors may influence the mother’s evaluation of the child’s pain and possibly her approach to the child’s pain management. This again underscores the need for a comprehensive approach to pain management and coping strategies directed towards children and their parents.

Another potential explanation for the high prevalence of reported use might be related to a general lack of knowledge about medication use within this age group. Some studies suggest that self-medication can begin as early as 11 or 12 years of age and significantly increases with age [14]. Consequently, the higher prevalence of recent use among children compared to adolescents might arise because children are not cognisant of the potential negative side effects of OTC analgesics and are to a lesser degree equipped to interpret label instructions. These medications are furthermore often easily accessible at home, and children might therefore have the misconception that such pain relievers are safe [36]. Easy access, limited knowledge and the misconception that OTC analgesics are safe further pose a potential risk of overdose.

Our results furthermore show that a higher frequency of participation in sports significantly increased the risk of recent use of OTC analgesics. Generally, research has demonstrated a positive influence of physical activity for mental well-being and health [37]. On the one hand, sport injuries cause pain, but such pain is normally more acute. On the other hand, it has been suggested that pain induced by training load could be a factor in adolescents’ more persistent pain and use of OTC analgesics [25]. While the effect size in our study is small, it is noteworthy that even within this young age group, a higher level of sports activity significantly elevated the risk of recent use of OTC analgesic, even when adjusted for pain in different parts of the body. There have been some concerns raised regarding the professionalisation of children’s sports in Norway, where the demands for activity and performance that were previously reserved for older age groups are now becoming prevalent in younger age groups [38]. Such high demands in sports performance in combination with high activity levels might have reached a tipping point regarding some children’s resources to handle stress.

In sum, our findings indicated that the use of OTC analgesics in childhood may meet needs other than treating mild to moderate physical pain and may possibly act as a placebo for coping with physical and emotional distress and negative life events. Such use indicates unhealthy coping strategies [32]. The childhood and early adolescent period are pivotal stages in the development of lifestyle habits. Behavioural patterns established in childhood are likely to persist into adulthood. Given this, the high prevalence of use, along with usage identified in this study beyond the recommended indicators, may pose a potential public health concern. The use of OTC analgesics might also mask underlying issues associated with various life stressors or psychosocial difficulties, potentially obstructing timely and appropriate help.

### Strengths and limitations

This study draws upon a large sample of children aged 10 to 12, drawn from the general population (*N* = 102,919), with a satisfactory response rate from across the country. Given the substantial number of participants, we regard this population as representative of Norwegian children. However, it is crucial to acknowledge the study’s limitations. Firstly, our data were cross-sectional, which precludes the drawing of causal inferences. Secondly, our outcome measure did not specifically inquire about OTC analgesics, but rather about ‘tablets for headaches or other pains’. Children may not always know the medications they receive from their parents, and could report taking other substances for pain, like allergy medication, dietary supplements or gastroesophageal reflux medication. This could inflate the reported usage. Consequently, the reported prevalence and associations of OTC analgesic use should be interpreted with caution and further substantiated in studies of similar samples, using a more standardised question about OTC analgesics. This caution is furthermore particularly pertinent given that part of the data collection occurred during and immediately after the COVID-19 pandemic. It is conceivable that the rate of OTC analgesic use was correlated with the pandemic. Additionally, the reopening of society could have heralded the resurgence of milder diseases, which may have affected OTC analgesic use. Moreover, as fever is one of the primary reasons for using OTC analgesics, it would have been beneficial to be able to control for this factor. Unfortunately, the questionnaire did not include information on whether respondents had experienced a fever in the past week. Therefore, the prevalence of OTC analgesic use needs to be confirmed in studies of similar samples under normal conditions and adjusted for fever. It would also be useful to have more specific information about the type of painkillers they used, given that different medications can have different side effects. Another limitation was the lack of information on whether the children in our sample self-medicated or consulted with an adult or health professional first. Our study’s measures were furthermore based on self-report, which may introduce some uncertainty, as children may struggle with accurately reporting certain details, such as their hours of sleep or screen time. Lastly, the measures used in this study assessed different time intervals. For instance, a child might have experienced pain several times in past month, but not necessarily in the last week, which is the time frame for the use of OTC analgesics. As such, their use of such pain medicine might not align with when they experienced the pain and may result in an underestimation of the relationship between pain and use of OTCs.

## Conclusions

To the best of our knowledge, this is the first study in a nationally representative sample of Norwegian children to systematically investigate the relationship between recent use of OTC analgesics and such an extensive set of factors, both within and beyond the range of indicators for use of the medication. Our findings showed that there was a high prevalence of recent use of OTC analgesics among children. Furthermore, our findings indicated that this use of pain medication was partly underpinned by factors outside the range of indicators for OTC analgesics. Although the strongest and most consistent relationship was found between recurrent headache and pain in different parts of the body, consistent results were found for different life strains, where depressive symptoms, having experienced repeated bullying and having a higher frequency of participation in sports were significantly related to an increased risk of recent use of OTC analgesics. As such, our findings indicated an inappropriate use of OTC analgesics among 10- to 12-year-olds. Increasing knowledge and skills regarding treatment of pain and coping with general life strains among children and their parents may improve health behaviours and prevent the negative effects of OTC analgesics use. Working against a general trend, where OTC analgesics are used to alleviate symptoms of psychosocial difficulties and psychological distress, could also have the potential to decrease the risk of overuse.
